# Touch DNA Sampling Methods: Efficacy Evaluation and Systematic Review

**DOI:** 10.3390/ijms232415541

**Published:** 2022-12-08

**Authors:** Pamela Tozzo, Enrico Mazzobel, Beatrice Marcante, Arianna Delicati, Luciana Caenazzo

**Affiliations:** Department of Cardiac, Thoracic, Vascular Sciences and Public Health, Legal Medicine Section, University of Padova, 35121 Padova, Italy

**Keywords:** touch DNA, genetic profile, crime scene, forensic genetics, systematic review

## Abstract

Collection and interpretation of “touch DNA” from crime scenes represent crucial steps during criminal investigations, with clear consequences in courtrooms. Although the main aspects of this type of evidence have been extensively studied, some controversial issues remain. For instance, there is no conclusive evidence indicating which sampling method results in the highest rate of biological material recovery. Thus, this study aimed to describe the actual considerations on touch DNA and to compare three different sampling procedures, which were “single-swab”, “double-swab”, and “other methods” (i.e., cutting out, adhesive tape, FTA^®^ paper scraping), based on the experimental results published in the recent literature. The data analysis performed shows the higher efficiency of the single-swab method in DNA recovery in a wide variety of experimental settings. On the contrary, the double-swab technique and other methods do not seem to improve recovery rates. Despite the apparent discrepancy with previous research, these results underline certain limitations inherent to the sampling procedures investigated. The application of this information to forensic investigations and laboratories could improve operative standard procedures and enhance this almost fundamental investigative tool’s probative value.

## 1. Introduction

When approaching a crime scene, given the limited availability of biological evidence, it is essential to choose the best forensic approach to collect DNA evidence in order to achieve as much information as possible. Among many possibilities, recovering DNA from different biological materials left behind by criminals and matching them to suspects has become increasingly relevant, giving an effective tool to investigators and courts. Moreover, in recent years, scientific improvements in recovery, extraction, amplification, and analysis led to obtaining informative profiles even from extremely limited traces [1,2,3,4,5,6]. In this scenario, the capacity to interpret DNA deposited through handling items (“touch DNA”) becomes a necessary tool in most forensic genetic laboratories, even if some challenges remain.

“Touch DNA” can be defined as DNA transferred from a person to an object via contact with the object itself. In the literature, this form of evidence has also been called “contact DNA”, “trace DNA”, or “transfer DNA”. The nature of this type of genetic material is still the subject of ongoing scientific debate, which expresses the lack of knowledge in the present forensic field. While many studies support DNA deposited by touch came from shed keratinocytes [7,8], several papers offer a wider perspective, identifying multiple sources as complete or partial skin cells, nucleated epithelial cells from other fluids or body parts in contact with one’s hands (i.e., saliva, sebum, sweat), or cell-free DNA, either endogenous or transferred onto the contact region from the abovementioned fluids [9,10]. In particular, cell-free DNA has been proven to be a reliable source of genetic material, often generating higher yields than its cellular counterpart [11] although considerable doubt remains about its origin; it is still unclear whether cell-free DNA is derived directly from body fluids or whether it is released after cellular degradation following touch deposition. Reports of fragmented DNA traces deposited from freshly washed hands suggest that DNA alteration begins within the organism [12].

However, touch DNA samples are generally known to contain low levels of DNA [13] and the presence of degraded genetic material, regardless of its origin, makes genotype detection challenging [14,15,16,17,18,19,20].

**Figure 1:**
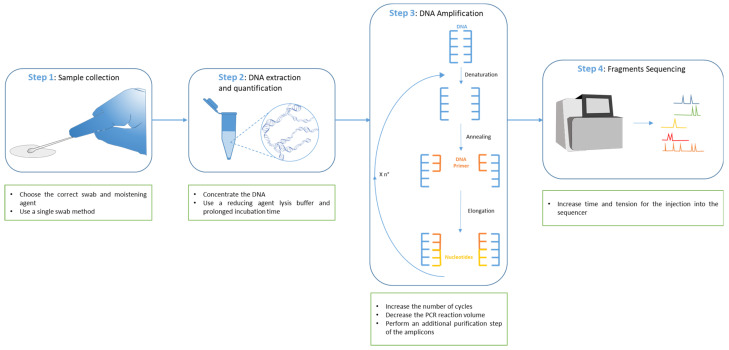
DNA analysis workflow and improvement for low template DNA. In sample collection, the correct swab should be chosen, and, in particular, collection through a single swab should be performed on non-porous surfaces; the use of tape lifting is a preferred option for porous surfaces. Moreover, in this step, the moistening agent is also of fundamental importance to improve the final results (Step 1). Other possible solutions to improve the DNA analysis of low template DNA consist of the concentration of the DNA after its extraction or in the use of reducing agent lysis buffer with a prolonged time of incubation to increase, in both cases, the concentration of the final extracted DNA in the reaction volume (Step 2). The following step of DNA amplification may be modified in different ways to improve the DNA analysis in the case of low-template DNA. It is possible to increase the number of PCR cycles, decrease the PCR reaction volume to further concentrate the amount of DNA, or perform an additional purification step of the amplicons (Step 3). Eventually, it is possible to also intervene in the last step of fragments sequencing by increasing the time and the tension for the injection of the DNA fragments into the sequencer (Step 4).

Degraded DNA is not the only component of touch deposits that can compromise forensic profiling. The presence of small amounts of genetic material available, sometimes even below the minimum thresholds of modern highly sensitive commercial STR kits, is another phenomenon commonly found in contact samples. In this contingency, PCR amplification can miss the detection of short DNA fragments even when the procedure is implemented with additional cycles to maximize the results. These evident limitations suggest the occurrence of stochastic effects related to sampling techniques rather than mere analytical defects [21,22] and precisely describe the so-called Low Template DNA (LT-DNA) or Low Copy Number DNA (LCN-DNA). In Figure 1 we describe methods used to enhance LT-DNA extraction, amplification, and sequencing.

Many factors can affect the quantity and the success of recovering the genetic material, schematically grouped into three categories of variables influencing sample generation, deposition, and analysis.

The concept of good or bad shedder status, primarily introduced in 1999 [23], is a person’s propensity to deposit a high or low amount of DNA on a touched object, respectively. According to the current notions, this ability varies greatly between individuals or in the same person under distinct conditions [24]. Although biological and genetic factors affecting this status are largely unknown, age, sex, and certain activities (i.e., touching DNA-free objects, wearing gloves, rubbing fingers on body parts) seem to influence the deposited traces. Generally, men shed more DNA than women, especially younger males compared to older ones (the trend was not investigated in females) and washing hands can reduce the available quantity [25,26]. In contrast, physical activities involving sweating leads to an increase in DNA transfer [27]. Closely related to this subject, body location impact results too, for example, sebaceous skin areas (vs. non-sebaceous), the dominant hand (vs. non-dominant), and fingertips (vs. palms) potentially facilitate DNA deposits [28].

Biological evidence can be virtually left behind everywhere during criminal activities, i.e., from wooden murder weapons to metallic handle doors. Considering this, in daily forensic practice, different material compositions had to be investigated, with variable results. Several authors have reported increased sloughed epithelial cells on rough and porous substrates, while non-porous substrates adhere to genetic material less readily [9,29]. Thus, fabrics and cotton appear to be better DNA collectors than plastic or glass surfaces and it has been proven more difficult to consistently recover touch DNA from metal surfaces [30]. The manner and duration of contact also influence the amount of genetic material transferred. It has been demonstrated that DNA deposits increase when pressure or friction are involved [28], directly proportional to the intensity applied [31]. Instead, the influence of time in the resulting amount of DNA on handling/wearing items remains controversial. While recent studies propose a linear correlation between variables [32], previous papers excluded any linkage, suggesting the origin of traces in a single transfer step upon initial contact [33]. Additionally, the possible interactions between other investigative methods, such as dactyloscopic enhancement methods, bloodstain enhancement methods, and DNA typing techniques, cannot be excluded [34,35,36].

Since each operative step expresses great availability in devices and techniques as well as in the manner of recovering, processing, and analysing samples, results from DNA analysis may be influenced by the combination between the singular forensic approach to the crime scene and following laboratory procedures [37,38]. Considered from a methodological perspective, the collection of touch DNA traces may involve the use of various sampling devices, such as swabs, adhesive tapes, or directly examining the evidence, in whole or in part. Considering their cost-effectiveness and minimal training requirements, the use of swabs is one of the most versatile and widely used methods. They can be applied dry or moistened with several agents and in varied materials. For example, standard cotton swabs are traditionally preferred for the collection of biological fluids and, notwithstanding further research, showed a tendency for the organic residue to get entrapped within cotton fibres, reducing sample availability [39,40]. When trace DNA is expected to be recovered, the double-swab technique [38,41] can be implemented. It consists of a wet swab and a second dry one sequentially applied onto the surface of interest, aimed at maximising recovery. Although the efficiency of this method has not been fully discussed, it is usually exploited to improve the collection of cellular material [42]. When other procedures are employed, effective alternatives are represented by “cutting out” the sampling area of soft tissues or the adhesive tape lifting the solid surface. The last sampling method is quick and straightforward, and tapes with better adhesion have been reported to produce a higher yield of trace DNA than swabbing, although the stickiness, rigidity, and size of the tape make the interpretation of the results more difficult [43,44,45,46].

Laboratory methods employed also affect the success of touch DNA analysis. Once recovered, standard workflows for processing touch DNA evidence first of all involves DNA extraction, for which a multitude of approaches exists, and then DNA quantification is conducted [47], which is critical to determine the quantity and quality of DNA extracted. This process is fundamental to decide the downstream genotyping methods to use and the proportion of the initial amount of evidence to submit to possible destructive analysis, thus, achieving a more informed interpretation of further analytical results [48]. However, the DNA extraction and quantification processes both result in the loss of a portion of the original sample and increase the probability of introducing exogenous DNA [49]. The amplification phase frequently implies the use of one of the commercially available kits most commonly used for criminal cases [50,51].

As can be inferred from the above, numerous factors influence touch DNA’s effectiveness as a forensic tool. Thus, we present here a brief review regarding the current state of knowledge on touch DNA analysis, with a particular focus on the impact the sampling techniques have on the results. The present paper evaluates several experimental settings in which different sampling methods have been used to provide valuable guidance in selecting the most appropriate collecting technique in relation to operative conditions. We believe it is necessary to enhance each analytical phase of the investigation in order to maximise the chance of finding useful profiles at crime scenes.

## 2. Materials and Methods

This review was performed in accordance with the Preferred Reporting Items for Systemic Reviews and Meta-Analyses (PRISMA) Guidelines [52].

In December 2021, a systematic literature review was performed by selecting papers from the Pubmed Database, according to the query “touch DNA”. The search terms were intentionally kept generic to include the highest number of potentially interesting works. A total of 997 articles were identified. Different inclusion criteria were then applied using specific PubMed filters to start the screening process: (1) English or Italian language; (2) availability of abstract and full text. Duplicates were manually removed. The screening process was conducted by the selection of titles and abstracts, and, when necessary, the evaluation of the full text. In cases of doubt, the consensus opinions of the research supervisors were solicited.

After title and abstract evaluation, a total of 136 manuscripts were considered. In the last phase, articles were selected when results were expressed in the form of STR alleles number (Group 1), informative profiles (Group 2), and percentage or DNA quantities (Group 3) to allow the comparison even between different experimental settings. Eventually, a total of 60 studies were carefully chosen.

**Figure 2:**
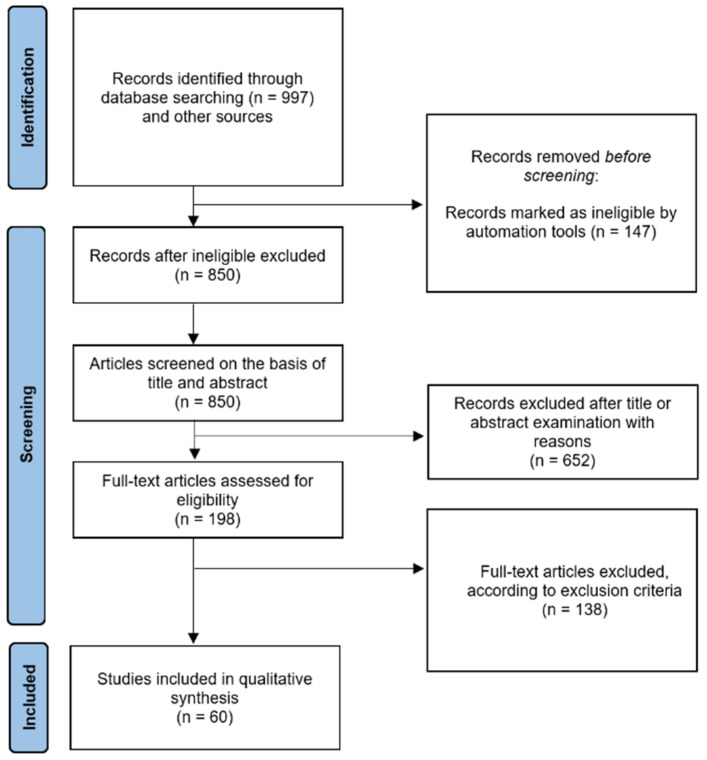
Preferred Reporting Items for Systemic Reviews and Meta-Analyses (PRISMA) 2020 flow diagram. A total of 60 studies were included in our systematic review.

The PRISMA flow chart in Figure 2 summarises the study screening and selection process as described above.

## 3. Results

### 3.1. STR Alleles and Informative Profiles

Based on the assumption that each article is composed of several separate tests, the experimental settings were highlighted (i.e., the number of samples collected, the recovery method, the extraction process, and the amplification procedure) to help distinguish the individual trials. Then, each trial’s results, represented by the mean number of STR alleles obtained, was converted into a percentage, compared to the specific amplification kit used, and classified as “low” or “high” if it was less than or greater than 66%, respectively. Similarly, the mean percentage of informative profiles was categorized as “low” or “high” with the same distinctive values.

**Figure 3:**
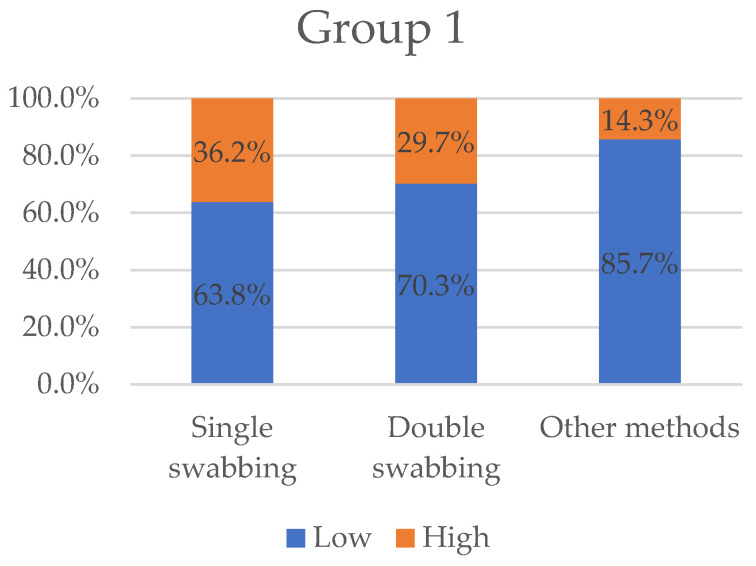
Variables “low” and “high” grouped by sampling methods for Group 1. With 36.2%, single-swabbing obtains the greatest “high” value, followed by double-swabbing (29.7%), and other methods (14.3%).

We eventually individuated 9 articles (15% of the total) in which the results were expressed as STR alleles obtained (papers shown in Table 1). Figure 3 displays the variables “low” and “high” grouped by three types of sampling methods (single-swabbing, double-swabbing, and other methods).

**Figure 4:**
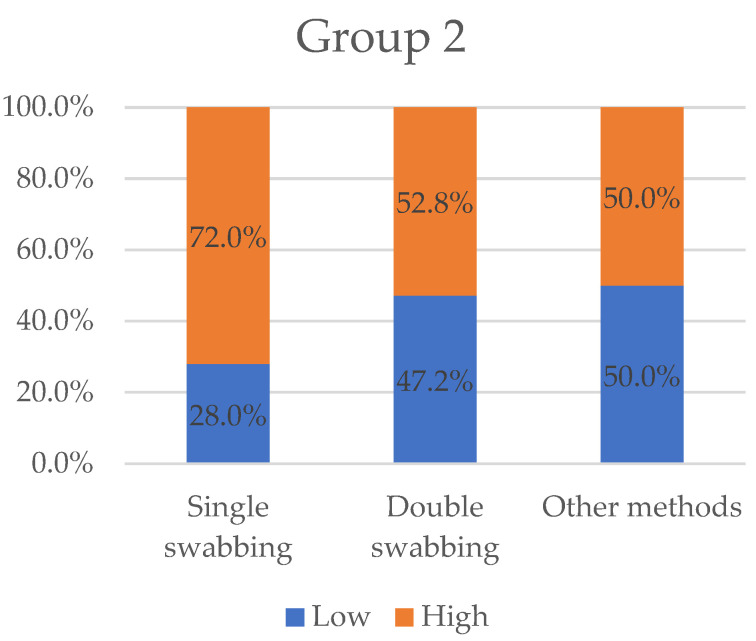
Variables “low” and “high” grouped by sampling methods for Group 2. Other methods collected the worst “high” value with 50%. Double-swabbing and single swabbing obtained 52.8% and 72%, respectively.

Likewise, 14 papers (23.4%) selected stated their results in the form of informative profiles (articles in Table 2). In Figure 4, we categorised the variables “low” and “high”, in percentage by the same previous sampling method type (single-swabbing, double-swabbing, and others).

### 3.2. DNA Quantitation

The last group of papers consisted of 43 articles where the authors published their results as DNA quantities, which represents 66.7% of the total. To be able to compare different findings, we identified two sub-groups: experiments where DNA concentration (Group 3a, with 17 articles) was declared, and trials where DNA quantity was indicated in absolute value (Group 3b, with 26 articles). Table 3 and Table 4 report the selection of the respective papers.

**Figure 5:**
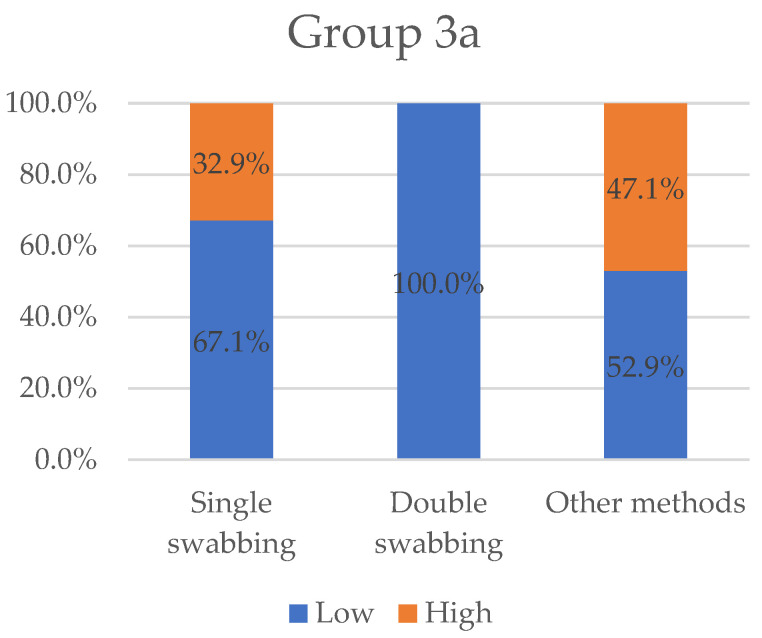
Variables “low” and “high” grouped by sampling methods for Group 3a. “Low” value represents the totality of results collected for double-swabbing. Single-swabbing and other methods obtained 67.1% and 52.9%, respectively.

**Figure 6:**
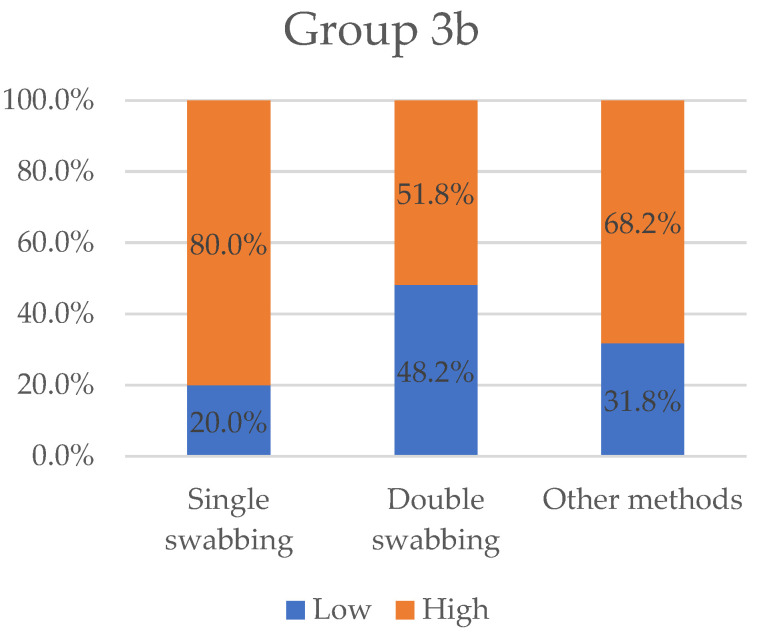
Variables “low” and “high” grouped by sampling methods for Group 3b. Single-swabbing appears to be the most efficient technique, with a “high” value equal to 80%. Other methods and double-swabbing collected 68.2% and 51.8%, respectively.

As for previous result types, we set cut-offs to classify the efficacy of different sampling methods. When the mean DNA concentration reported was under or above 0.1 ng/uL, a “low” or “high” value was assigned, respectively; the same variables were attributed when mean DNA quantity resulted in less than or greater than 1 ng. Figure 5 and Figure 6 show the values, in percentage, grouped by sampling methods (single-swabbing, double-swabbing, and other methods).

## 4. Discussion

The collection and analysis of touch DNA, especially when low amounts of genetic material are expected, can be challenging yet extremely precious for investigations. Touch DNA testing is limited by the difficulty of obtaining not only sufficient quality DNA to generate a complete profile, but also sufficient material to allow re-testing. Hence, optimising the procedures is fundamental even to improving the STR typing success rate. Moreover, studies investigating touch DNA often implement wide variability among experimental settings, with few papers examining the topic transversally. This analysis was designed to operate a literature review on touch DNA, with a focus on the comparison between the efficacy of different sampling methods. Since there is significant variability in the way results are presented and on what kind of data the comparison of touch DNA scenarios is based, we evaluate the performance of three collecting technique categories (single-swabbing vs. double-swabbing vs. other methods) by analysing the mean number of STR alleles, the percentages of informative profiles, and the quantity of touch DNA obtained. This variability in results can partially be explained by the fact that there is currently no consensus regarding which aspects of analysis are most suitable for comparing DNA traces [28]. DNA quantities seem ineffective, from an investigative standpoint, as they do not correlate with profile quality and do not contain any information about the presence of more than one contributor. However, they can provide an insight into the efficacy of procedures, the aim of the present study, and assist in the interpretation of research findings [102]. On the other hand, some experimental studies evaluate outcomes by analysing profile compositions. This sub-group was also considered to provide a broader perspective on the topic.

### 4.1. Single-Swabbing

In general, swabbing appears to be the most common procedure used, with other methods being applied depending on the setting. A large majority of the trials (72.6%) were conducted using a swabbing technique, as compared to only 27.4% of experiments that applied alternative approaches. From the examination of the results, single-swabbing emerges as an effective sampling technique, with the greatest percentage of “high” efficiency in Group 1 (36.2%), Group 2 (72%), and Group 3b (80%). In Group 3a (32.9%), however, its effectiveness appears as the second-best value. A possible explanation for the current considerations could be its extreme versatility. Swabs vary in several ways, such as the material from which they are made, their thickness and length, how tightly they are wound and/or articulated with the swab shaft, the shape and design of the storage/transport tubes, and the inclusion of or not of features that help to preserve the DNA, such as vents for improved air-drying, desiccants, or antimicrobial chemicals [103]. To maximise the chance of obtaining an informative DNA profile, swabs can be moistened with fluids such as sterile water and laboratory or commercial detergents [104]. Thus, crime scene officers have the possibility to adapt the most efficient combination, both regarding the substrate from which the sample is being collected and the type of biological material.

### 4.2. Double-Swabbing

Scrubbing an area with multiple swabs (and the co-extraction of these tools) has been promoted to enhance the overall recovery of trace DNA. It has now become a common practice, since some evidence stated a single moist cotton swab picks up less than half of the available sample [105]. In the present work, we found a controversial performance of the technique, as it did not achieve the best result in any of the groups considered. All the experiments in Group 3a produced a low value of DNA traces. Given the limitations of the present statistical analysis, it seems to be in direct contradiction to previous works showing that this procedure is recommended and improves the quality of the resulting DNA profiles [38,41,103]. Actually, De Bruin et al. [106], in comparing the double-swab method versus stubbing (an adapted tape-lifting technique) for collecting offender epithelial material, underline its slightly better performance despite not being as easy a procedure. Moreover, Vickar et al. [107] found that M-Vac^®^ (Microbial Vacuum), an industrial device initially developed to sample food for potential pathogens, was better performing than double-swabbing for touch DNA collection on brick surfaces, even if it collected less DNA on non-porous tiles. As it is evident, the double-swab method does have limitations, particularly when used on certain substrates that can be found at crime scenes. According to this, the present considerations cannot exclude the possible influence of the adequacy with which the sampling procedure has been implemented in each trial. Under non-optimal experimental conditions, the double-swab technique not only yields less DNA than alternative methods, but it also damages the surface of items [44]. The success rate of obtaining a DNA profile from contact traces is largely dependent upon the selection of the appropriate recovery method for biological material and how it is applied.

### 4.3. Other Methods

In this last group, several procedures have been proposed in the literature. Overall, this category results in the most effective tests in Group 3a (47.1%) and the second-best in Group 2 (50%) and Group 3b (68.2%). In Group 1, this category collects the worst rate, with “low” efficacy (85.7%). The most frequently used sampling method examined in the present group is the so-called tapelifting, which consists in repeatedly pressing the adhesive part of a strip against the material surface of interest. Many other studies have already investigated its efficiency. Barash et al. [108] found that the tape collection of biological material simplifies sampling, is non-destructive, and is also highly effective in genotyping DNA from many previously untested items left at crime scenes. Another work evaluates nine collection methods in sampling touch DNA from human skin following skin-to-skin contact in mock assault scenarios [53]. The results express that the different tools did not have a distinct impact on the STR recovery even if adhesive tape seemed to be the least adequate for this purpose as it achieved the lowest DNA collection. Surprisingly, FTA paper scraping was employed in several experiments, while just a few papers exist in the forensic literature. It employs a novel approach based on Whatman FTA cards^®^ that was used to collect touch DNA from the steering wheel surface in one case study [109]. Based on Kirgiz et al.’s work [56], FTA paper scraping seemed to yield significantly more DNA when compared to double-swabbing and tapelifting. The authors also provide some possible explanations for these concerns. In particular, FTA paper chemical composition allows greater preservation and release of DNA, a larger sampling area than swabs and a slower drying process. The “cutting out” technique is another procedure engaged in the considered articles. Despite some critical constraints, such as the material on which it is implemented (not every surface can be cut out) and its irreversibility, it has been reported to achieve the best results in DNA recovery in comparison with adhesive tape and dry swabbing [42]. Despite the limitations of a global consideration, these alternative collection procedures seem to be available in limited experimental groups, as evidenced by the low number of trials. These restrictions may also account for the unsatisfactory outcomes of the present paper regarding the efficacy of the treatment. It is likely that challenging scenarios requiring unconventional approaches may produce low-quality DNA samples because of the intrinsic complexity rather than the ineffectiveness of the recovery methods.

From our perspective, single-swabbing appears as an effective first-level technique, due to its versatility, cost-effectiveness, and ease of use. Virtually, this tool can be applied to every type of solid surface, with different biological matrices and high efficiency, as our study suggests. In the case of a limited number of evident traces, this collecting method may be preceded by visualisation techniques or by moistening the device to enhance the recovery success. When operative settings are particularly challenging, i.e., insufficient availability of samples or dryness of specimen, double-swabbing may be implemented as a second-level technique. However, the surface material needs to be carefully chosen, as the procedure has shown low efficacy when applied to porous patterns. Lastly, alternative methods represent dynamic forensic tools that may be used as third-level procedures in certain circumstances. In particular, the use of tapelifting is limited by a subsequently more complex extraction process and low performance on the human skin surface. FTA paper scraper seems to be a promising collecting method, which undoubtedly requires further investigations into its recovery rate on different materials. When touch DNA samples need to be recovered from soft tissue with great availability of evidence, direct cutting appears as a valid solution, even compared to traditional swabbing.

In conclusion, evident limitations underline our review, which are intrinsically related to the difficulty of the subject matter. Firstly, as a complete and systematic review requires, we consider an extensive temporal range to collect a significant number of experiments. Nonetheless, the number of articles taken into consideration may still be insufficient. Unfortunately, results from older studies must be treated with caution when compared to more recent publications. This is because the sensitivity of detecting traces of DNA has increased appreciably in recent years, potentially adulterating the final reflections. Secondly, besides sample collection, DNA profiling success is dependent on extraction technique, quantification method, and amplification procedures. These considerations are certainly complicated by inter-laboratory and inter-individual differences regarding profile assessment and internal standard practices. Since it is not feasible to consider every contribution, we assume each trial has been conducted according to the most appropriate, yet internationally validated, available procedures. There is no doubt that further analysis of touch DNA variables influencing outcomes will contribute to shedding light on a still-controversial topic.

## 5. Conclusions

The collection of useful touch DNA evidence cannot prescind the selection of an appropriate sampling method. While the current scientific opinion on the topic remains questioned, this review contributes to the debate by offering an updated perspective on the actual state of the art. While single-swabbing appears more efficient than alternative methods, double-swabbing does not improve touch DNA collections in advance. Less common sampling procedures such as FTA paper scraping, cutting out or adhesive tape-lifting require pre-operative considerations to maximise their unquestioned efficacy. The present paper also highlights some intrinsic limitations, such as the inevitable impact of numerous variables on outcomes. Among these, the site on which biological material sampling is conducted and the type of traces recovered result as the most significant. Different settings require different devices to obtain the highest profiles from touch DNA samples. This information, along with future considerations, will contribute to enhancing the forensic ability to produce interpretable DNA profiles during investigations, even when minimal biological traces are available, with potential benefits to the criminal justice process.

According to the studies examined in this review, it is nowadays possible to obtain satisfactory results from the analysis of LCN-DNA, depending on the recovery technique used. However, almost all articles revealed that further research is needed on the impact of using different methodologies to collect samples to determine the most effective collection method. More comprehensive knowledge of detecting a profile based on the type of object and its history, identifying the most appropriate area(s) to target for DNA sampling, and the impact of additional factors, such as duration, frequency, and manner of contact, is required. Additionally, further research regarding the mechanisms of DNA shedding status, including the differences between sexes, the effects of activities performed before deposition, as well as other factors that may affect the amount of DNA deposited, is highly desirable for the forensic discipline. Being able to know, harmonise, and improve these aspects would definitely strengthen the value of DNA evidence in courtrooms.

## Figures and Tables

**Table 1:** Papers categorized in Group 1. Features displayed are authors and publication year, number (n°) of samples collected, sampling methods implemented, important findings, and remarks highlighted.

Authors	Samples n°	Sampling Methods	Important Findings	Remarks
Kallupurackal et al., 2021 [53]	180	Single-swabbing, double-swabbing, adhesive tapes	Results indicate COPAN FLOQ^TM^, double-swab technique and regular swabbing techniques with cotton swab performed equally well across all tested methods.	Results could be retested and confirmed by selecting some of the best-performing methods and taking a larger number of samples per method in a future study.
Meixner et al., 2020 [54]	67	Single-swabbing	It is possible to obtain a complete DNA profile from both blood stains and touch DNA on skin specimens immersed in water even after several days, depending on the aquatic environment.	Samples immersed in water hold potential for the forensic identification of an offender who has left touch DNA or blood stains on the victim.
Hefetz et al., 2019 [55]	240	Double-swabbing, adhesive tapes	Deposition pressure significantly influenced the size of the developed fingermark, their quality, and the number of the amplified STR loci and forensically useful DNA profiles recovered.	When collecting fingermarks from donors excessive deposition pressure should be avoided, otherwise the processed impressions might appear blurred.
Kirgiz and Calloway, 2017 [56]	140	Swabbing, adhesive tapes, FTA paper scraping	In particular cases, there may be enough touch DNA on the steering wheel of vehicles to yield a complete STR profile of the last driver.	DNA collected from steering wheels using FTA paper is more likely to result in a more complete STR profile compared to swabbing or tape lifting.
Tonkrongjun et al., 2019 [57]	50	Single-swabbing	Combining the staining process with direct STR amplification resulted in more alleles being recovered from mock improvised explosive device (IED) evidence.	Fluorescence level directly correlated with the number of alleles obtained, suggesting that the dyes can be used to locate areas with higher concentrations of touch DNA.
Thanakiatkrai and Rerkamnuaychoke, 2019 [58]	270	Single-swabbing	Direct PCR should be considered for processing bullet casings. In mock casework experiments to mimic real-world gun sharing, direct PCR mainly picked up the alleles of the person who loaded the bullets.	The use of direct PCR with touch DNA from bullet casings detected more alleles than DNA extraction.
Baechler, 2016 [59]	1236	Double-swabbing	Results provide useful information for decision-making and prioritisation at the crime scene, at the triage step, and insights for DNA database managers and users.	Whatever the operational context, better-informed decisions contribute to enhance resource allocation and the efficiency of forensic science efforts.
Horsman-Hall et al., 2009 [18]	292	Double-swabbing	The Plexor^®^ HY System results proved DNA recovery to be sufficient for STR typing. When testing samplings of individuals handling shotshells only as necessary for firing, no significant difference was observed when comparing results obtained from the PowerPlex1 16 BIO and Minifiler^TM^ kits.	Data does not support PCR inhibitors being present in the majority of shotshell case samples, but poor STR amplification results in shotshell cases are more likely due to DNA damage, possible degradation, and/or low-level DNA.
Schwender et al., 2021 [60]	168	Single-swabbing	The shedder test results and data ranges were comparable to those of other shedder tests. This study identified moisturisers as a novel factor influencing proposed shedder statuses and corresponding DNA transfer.	To address activity-level hypotheses or questions during legal proceedings, transfer studies with high and low DNA depositors could be executed to encompass a range of possible transfer outcomes.

**Table 2:** Papers categorized in Group 2. Features displayed are authors and publication year, number (n°) of samples collected, sampling methods implemented, important findings, and remarks highlighted.

Authors	Samples n°	Sampling Methods	Important Findings	Remarks
Kanokwongnuwut et al., 2021 [61]	100	Swabbing, adhesive tapes	Tapelifting is unsuitable for cell-free DNA collection from non-porous surfaces and only facilitates the collection of corneocytes, which carry a lower amount of DNA.	Where no alternative to tapelifting exists, it is recommended processing the samples through direct PCR; this approach requires ≥4000 visualised corneocytes for the generation of a full DNA profile.
Al-Snan, 2021 [62]	5	Swabbing, adhesive tapes, direct cutting	Proper handling of RDX-C4 samples is needed. Many acceptable and fit STR profiles were generated using the techniques mentioned in the study.	Collecting DNA from the RDX-C4 sample will give a forensic lead to directly identify the suspect(s) who manufactured the improvised explosive device (IED).
Hefetz et al., 2019 [55]	240	Double-swabbing, adhesive tapes	Deposition pressure significantly influenced the size of the developed fingermark, their quality, and the number of the amplified STR loci and forensically useful DNA profiles recovered.	The authors suggest that when collecting fingermarks from donors one should avoid excessive deposition pressure, otherwise the processed impressions might appear blurred.
Francisco et al., 2020 [63]	104	Double swabbing	The Casework Direct Kit showed better efficiency for processing touch DNA samples, enhancing the chance of recovering deposited DNA and improving STR profile quality when compared with DNA IQ.	Limitations on the quantification step for these samples with a low quantity of DNA were highlighted. More studies are necessary to compare quantification kits using samples extracted with casework.
Martin et al., 2018 [64]	312	Double-swabbing	The STR kit employed for amplification impacts the quality of the DNA profile obtained. Findings further demonstrate the success of direct PCR to enhance the STR profiles from touch DNA.	With some restrictions, Identifiler^®^ Plus should be used in preference of GlobalFiler^®^ for the amplification of touch DNA samples.
Kanokwongnuwut et al., 2019 [65]	24	Double-moistened swabbing	Touch DNA can be visualised after fingermark enhancement has been performed. DNA profiles were obtained from treated marks except after cyanoacrylate treatment.	For plain and un-patterned surfaces, the Diamond™ Dye fluorescence can be seen in ambient light, and this will be convenient for application at crime scenes.
Falkena et al., 2018 [66]	100	Single-swabbing	The correlation between the autofluorescent signal and DNA concentration in fingermarks was too weak to predict their DNA content.	The autofluorescent signals of fingermarks are not able to guide the forensic investigator reliably to fingermarks with a considerable DNA content.
Sołtyszewski et al., 2015 [67]	120	Single-swabbing	There was no significant difference between the amount of DNA deposited by male and female contributors.	When using AmpFlSTR^®^ NGM™, it is recommended to increase the number of PCR cycles from the standard 30 to 34 to boost the typeability of LT-DNA samples.
Templeton and Linacre, 2014 [68]	170	Double-swabbing	The authors demonstrate the ability to generate informative DNA profiles from latent fingermarks deposited by touch.	By eliminating the need to increase the PCR cycle number or concentrate the amplified products, the procedure described is easily adapted into working practices.
Romano et al., 2019 [69]	12	Adhesive tapes	This study illustrates the possibility to type DNA from fingerprints archived several years ago under uncontrolled conditions.	Contamination of the fingerprint represents a factor interfering with correct genotyping, rendering the interpretation of mixed profiles ambiguous.
Ip et al., 2015 [70]	76	Double-swabbing	QIAcube, QIAsymphony, and IQ all yielded extracts with a higher success rate for the subsequent DNA typing analysis, as opposed to Chelex and Blood Mini even after their concentration with Microcon.	The use of serially diluted blood and buffy coat samples, as well as the simulated touch DNA samples, could shed light on the effectiveness of these extraction methods on DNA analysis.
Subhani et al., 2019 [71]	72	Adhesive tapes	DNA profiles can be recovered from fingerprints, both groomed and natural, enhanced, and lifted using some of the most common powder/lift combinations.	Profiles obtained from fingerprint lifts are used as an intelligence tool to supplement the investigation rather than for identification.
Phipps and Petricevic, 2007 [72]	60	Double-swabbing	The success rate of obtaining a trace DNA profile on forensic casework items will depend on both the characteristics of the DNA contributor and the specific activities performed by the contributor before touching the item.	This study sheds some light on the variables affecting transfer DNA, such as the time since a person last washed their hands and which of the two hands an item is touched with.
Templeton et al., 2017 [73]	160	Single-swabbing	Direct PCR generates meaningful DNA profiles from powdered fingerprints, speeds up the processing of samples, and minimises contamination. Powders tested did not inhibit the direct PCR amplification.	However, DNA quantification of the sample cannot take place and there is no opportunity to remove potential PCR inhibitors.

**Table 3:** Papers categorized in Group 3a. Features displayed are authors and publication year, number (n°) of samples collected, sampling methods implemented, important findings, and remarks highlighted. N.A. not assigned.

**Authors**	**Samples n°**	**Sampling Methods**	**Important Findings**	**Remarks**
Sessa et al., 2019 [42]	240	Swabbing, adhesive tapes, direct cutting	The presence of a single DNA profile or the major contributor to a mixture obtained by sampling worn garments may not necessarily belong to the wearer.	Further knowledge of the frequency of detection of wearer and/or handler DNA profiles is required.
Oldoni et al., 2016 [32]	234	Double-swabbing, direct cutting	A large proportion of samples was characterised by the presence of unknown “background” alleles; indirectly transferred DNA is most often detected as partial/full minor DNA profile and less frequently as full major profile, whereas first and second users can provide major/minor autosomal STR profiles.	Further studies should explore both sets of porous and non-porous substrates, variable manner of contact, shorter experimental periods, longer time between DNA deposition and sample collection, and sample exposure to real casework conditions.
Comte et al., 2019 [8]	360	Single-swabbing	DNA seemed to remain stable after the time intervals, except when using the COPAN 4N6FLOQSwabs™ treated with an antimicrobial agent (crime scene variety), which resulted in significant DNA degradation.	Other combinations of the processes tested may provide good results elsewhere. However, findings from the different steps of this project may be useful or inspirational for other practitioners.
Hefetz et al., 2019 [55]	240	Double-swabbing, adhesive tapes	Deposition pressure significantly influenced the size of the developed fingermark, their quality, and the number of the amplified STR loci and forensically useful DNA profiles recovered.	When collecting fingermarks from donors, excessive deposition pressure should be avoided, otherwise the processed impressions might appear blurred.
Jansson et al., 2020 [74]	4	Single-swabbing	A sampling protocol for cartridge cases applying nylon-flocked swabs was developed. It was found that the material of the cartridge case, as well as the type of firearm, have a substantial impact on DNA yield.	It was not possible to take full advantage of the elevated DNA yield given by nylon-flocked swabs. Still, the number of usable STR profiles increased, but remained unchanged for cartridges.
Templeton and Linacre, 2014 [68]	170	Double-swabbing, single swabs	The authors demonstrate the ability to generate informative DNA profiles from latent fingermarks deposited by touch.	By eliminating the need to increase the PCR cycle number or concentrate the amplified products, the procedure described is easily adapted into working practices.
Forsberg et al., 2016 [43]	N.A.	Adhesive tapes	The introduction of the developed direct lysis protocol reduced the amount of manual labour by half and doubled the potential throughput for tapes at the laboratory. The reduction in pipetting steps and sample transfers lowers the contamination risk.	Differences in number of single-donor profiles and mixtures are related to differences in the sampled material rather than the tape-type or extraction procedure.
Tasker et al., 2017 [75]	83	Single-swabbing	DNA identification was equally successful when DNA was recovered from the end caps or the pipe shaft of PVC pipe bombs. However, the majority of STR profiles were of poor quality.	Heterozygote peak height imbalance and allelic drop-out were frequently observed, highlighting the difficulties of recovering DNA and generating reliable STR profiles from low-template and moderately degraded samples.
Parsons et al., 2016 [76]	N.A.	Double-swabbing	Through a predetermined examination strategy, it is possible to obtain both DNA profiling results and document examination findings, maximising the evidentiary value of these analyses for document exhibits.	This collaborative testing strategy could be extended to include fingerprint analysis. If successful, this would then allow fingerprint evidence to be recovered along with DNA and document examination evidence.
Tobe et al., 2011 [77]	N.A.	Adhesive tapes	Obtaining human DNA profiles from touched areas of animal carcasses could be rapidly implemented in laboratories already undertaking low-template DNA casework.	Future work is required to determine after which PMI (post-mortem interval) it would be impractical to analyse poaching remains.
Sewell et al., 2008 [78]	N.A.	Direct cutting	It was found that certain paper-types interfered with the successful extraction of DNA. Conversely, others allowed greater recovery of transferred DNA.	Whilst Low Copy Number DNA profiling increased the average percentage of the profile obtained, a higher incidence of PCR artefacts and contamination were observed.
Ostojic et al., 2014 [79]	700	Single-swabbing	It is difficult to obtain full STR profiles from single fingerprints reliably, but improvements are possible with different extraction methods and amplification kits and protocols.	Shedding score alone was not a reliable predictor of profile quality, because many deposited cells of a fingerprint may not be nucleated.
Oldoni et al., 2017 [80]	N.A.	N.A.	The DIP-STR markers perform well on challenging casework DNA samples containing low total DNA or high major/minor DNA ratio, irrespective of the sex of the DNA contributors and when paternally related males are involved.	More research on specificity and sensitivity thresholds beyond previously tested conditions, multiplex markers development, and further development of the statistical framework are needed.
Pang et al., 2007 [38]	40	Single-swabbing	The study presents a swabbing protocol for collecting trace DNA samples, which should improve the recovery of DNA from the crime scene exhibits. It also helps in standardising the swabbing protocol and preventing DNA contamination.	DNA profiling results can be improved by pooling the first wet and the second dry swabs together for extraction.
Yudianto et al., 2020 [81]	4	Single-swabbing	Property (cell phone and watch) swabs can be used as alternative materials in forensic identification using touch DNA analysis.	For adequate visualisation of the results, sufficient levels and purity of the DNA are needed.
Giovanelli et al., 2022 [82]	108	Single-swabbing	Success in DNA recovery is influenced by the type of swab used and by the shedder status. The PurFlock^®^ swab was more efficient for recovering donor alleles than the others	The study highlights the need to assess different materials and methods of collection of biological samples, considering collection, extraction, and amplification.
Moore et al., 2021 [83]	90	Double-swabbing, direct cutting	Informative DNA profiles were successfully obtained from both unfired and fired cartridges. Mixtures of DNA were observed from most cartridges, suggesting indirect transfer of DNA to the cartridges via the hands.	Further work is required to assess the impact of direct lysis and the mechanical agitation employed during sample lysis, as well as on firing and striation marks often examined on spent ammunition.

**Table 4:** Papers categorized in Group 3b. Features displayed are authors and publication year, number (n°) of samples collected, sampling methods implemented, important findings, and remarks highlighted. N.A. not assigned.

Authors	Samples n°	Sampling Methods	Important Findings	Remarks
Stoop et al., 2017 [84]	36	Single-swabbing, adhesive tapes	Data demonstrates that SceneSafe Fast™ Mini-tape sampling of touch DNA in combination with organic solvent extraction is more efficient than touch DNA sampling by swab.	The authors point out the importance of choosing the right extraction method, as conclusions need to be restricted to the tested cotton tissue.
Lim et al., 2016 [85]	16	Single-swabbing, adhesive tapes	The double-swab technique and mini-taping are equally viable choices for the recovery of touch DNA from cables. The enhancement allows for targeted recovery of DNA with more full profiles obtained.	Wet powder suspensions revealed disadvantages in their application procedures resulting in less DNA yields, poor profiles, and contamination issues.
Kirgiz and Calloway, 2017 [56]	140	Swabs, adhesive tapes, FTA paper scraping	In particular cases, there is enough touch DNA on the steering wheel of vehicles to yield a complete STR profile of the last driver.	DNA collected from steering wheels using FTA paper is more likely to result in a more complete STR profile compared to swabbing or tape lifting.
Dong et al., 2017 [86]	156	Double-swabbing	Greater amounts of DNA and number of alleles were detected on the porous substrates. The direct cutting method displayed advantages for porous substrates and the vacuum cleaner method was advantageous for non-porous substrates.	Although different pre-processing methods have a significant impact on the detection of touch DNA samples, the choice of the extraction method after pre-processing of the sample also plays a vital role in the examination of the sample.
Jansson et al., 2022 [24]	41	Single-swabbing	In many cases, the majority of DNA deposited on items and surfaces does not originate from the hands themselves but may have been transferred to the hands by touching, rubbing, or scratching other body parts or handling personal objects.	The strong association to facial DNA accumulation suggests that physiological mechanisms rather than differences in personal habits dictate individual shedder status.
Goray et al., 2020 [37]	143	Double-swabbing, single-swabbing	The findings may assist in assigning probabilities to DNA-TPPR events in cases where a person has temporarily occupied another environment.	More research is needed to ascertain the impact of using different methodologies (from collection to profiling) and to generate data to help determine frequency estimations for different types of profiles.
Daly et al., 2012 [29]	300	Adhesive tapes	In terms of DNA transfer and recovery, wood gave the best yield, followed by fabric and glass. There was no significant difference between the amount of DNA transferred by male or female volunteers.	In routine casework, a low-level DNA quantification result (less than 0.03 ng/μL of DNA) can be used as a cut-off point in deciding whether or not to profile certain samples.
Boyko et al., 2020 [87]	142	Double-swabbing	DNA of known recent passengers, close associates of the driver, and unknown individuals was collected. These findings may assist in sample-targeting within cars and the evaluation of DNA evidence.	The data on the types of profiles collected and who are contributing sources, given the known histories of the cars and their occupants, may assist those addressing questions regarding the presence and activities of a specific individual.
Ruan et al., 2018 [88]	300	Adhesive tapes	The transfer of foreign DNA onto an individual’s external clothing during a regular day is commonplace. Extraneous DNA may have been present on the clothing item prior to being worn and may have been transferred during laundering.	Further studies which examine ‘background’ DNA acquisition, are recommended to gain a better understanding of the mechanisms that lead to the transfer of trace DNA.
Al Oleiwi et al., 2017 [89]	40	Double-swabbing	The ability to recover DNA from samples treated with this infrared fluorescent powder highlights the minimally invasive nature of this fingerprint visualisation process, which when coupled with its inherent optical properties, provides the investigator with an extremely powerful tool.	Untreated latent fingermarks resulted in higher human quantification and relative fluorescent unit (RFU) values than samples treated with the powder alone. The inherent properties of the infrared fluorescent fingerprint powder allow for contrast in samples that would otherwise be very difficult to detect and treat for fingerprints.
Lacerenza et al., 2016 [90]	120	Single-swabbing, adhesive tapes	Transfer of cellular material different from the skin may underlie the occasional recovery of quality STR profiles from handled items. Gender may represent an important factor influencing the propensity of individuals to carry and transfer DNA through hand contact.	Further work, including an analysis of larger and more diverse experimental samples, as well as a study of the DNA/RNA transfer and persistence after different types of contact, is necessary to better support “activity level” inferences.
Bowman et al., 2018 [91]	266	Double-swabbing	Sampling from clothing worn over the assaulted area may be a better avenue for the recovery of the offender’s DNA post-assault where there has been significant time between assault and sampling.	The sampling from clothing requires further investigations to increase the accuracy of the probabilities of the LR of alternative scenario propositions.
Bonsu et al., 2021 [92]	N.A.	Single-swabbing, adhesive tapes	The study reinforces the previous finding of improved efficiency of trace DNA recovery from problematic metal surfaces utilizing the Isohelix™ swab moistened with isopropyl alcohol in contrast to a rayon swab moistened with water.	Further research on the impact of cautionary measures taken against the spread of infections in a pandemic situation on touch DNA transfer and persistence, and the recovery efficiency and the integrity of recovered DNA and STR profiles generated is required.
Sterling et al., 2019 [93]	20	Single-swabbing, adhesive tapes	The combined DNA extraction/protein trypsin digestion assay was able to generate full DNA STR profiles. Combining DNA and protein polymorphism maximises the information that can be gained from contact traces.	Further work is needed to identify reliable genetically variant peptide (GVP) markers, address background protein levels, and work on mixture detection and interpretation.
Butcher et al., 2019 [94]	36	Adhesive tapes	DNA from the second user of regularly used knives is detectable even after 2 sec of use. Removal of regular user DNA by a second user can impact proportional profile contributions. The proportion of indirectly transferred DNA is generally lower than directly transferred DNA.	Caution should be taken when relying solely on absolute quantities of DNA to inform evaluative interpretations, and other parameters, such as profile quality and relative contributions to mixed profiles, should also be considered.
Dierig et al., 2019 [95]	N.A.	Single-swabbing	Staining of bio-particles is only necessary for use in single-shed skin flake collection. However, it is proposed to prefer the swabbing of small areas over single-shed skin collection to largely avoid mixture generation and improve DNA yield.	Evolving biostatistical evaluation tools using continuous statistic models, such as EuroForMix, GenoProof Mixture 3, or STRmix™, might help to enable better separation of contributor profiles.
Oldoni et al., 2016 [32]	234	Double-swabbing, direct cutting	A large proportion of samples was characterised by the presence of unknown “background” alleles; indirectly transferred DNA is most often detected as partial/full minor DNA profile and less frequently as full major profile, whereas first and second users can provide major/minor autosomal STR profiles.	Further studies should explore both sets of porous and non-porous substrates, variable manner of contact, shorter experimental periods, longer time between DNA deposition and sample collection, and sample exposure to real casework conditions.
Solomon et al., 2018 [47]	2600	Double-swabbing, single-swabbing, direct cutting	Viable DNA is available in some archived latent fingerprint samples, and it can be retrieved for DNA profiling.	The addition of a post-amplification purification step fails to improve the STR profiles obtained from these samples and the increased sensitivity is more likely to intensify the presence of artefacts that further complicate data interpretation.
Bathrick et al., 2022 [96]	144	Double-swabbing	The number and type of fingerprint development treatments that are used can negatively impact the ability to obtain DNA from fingerprints.	Although the selection of appropriate development treatments can minimise the opportunities for DNA loss and damage, the development of CODIS-eligible DNA profiles is not guaranteed due to the variable amounts of DNA contained within fingerprints.
Goray et al., 2016 [97]	240	Double-swabbing	Shedder categorisation may be limited to the palm and the fingers of the hand and have relevance only to hand-touched surfaces and items.	Further research is needed to determine the shedder status of a DNA sample collected from casework-related items of interest.
Breathnach et al., 2016 [98]	N.A.	Adhesive tapes.	On worn garments, the probability of observing reportable DNA profiles is 61.9%. The wearer was detected as a single profile or part of a mixed profile in 50.8% of samples. When the wearer was present in a mixture, he was always observed as the major contributor.	Greater knowledge of the frequency of detection of reportable wearer DNA and/or toucher allows scientists to evaluate the likelihood of observing a matching profile if an individual wore a garment rather than touched it in disputed case scenarios.
Kita et al., 2008 [99]	6	Single-swabbing	Small amounts of fragmented DNA may be constantly sloughed off the cornified layers and sweat may contain the fragmented DNA. Therefore, it is conceivable that a genetic profile might be retrievable from any object touched.	Electron microscopic analysis showed the presence of small pieces of fragmented DNA on the cotton swabs. Therefore, the DNA on the swabs must have originated from skin tissue and become fragmented.
Van Oorschot et al., 2014 [100]	120	Double-swabbing, adhesive tapes, direct cutting	The degree of persistence of DNA from a prior user of an object depends on the type of object, the substrate it is made of, the area of the object targeted for sampling, and the duration and manner of contact by a subsequent user.	Greater knowledge of persistence will inform investigators regarding the likelihood of detecting a profile of a particular individual and assist with identifying the best area(s) of an object to target for DNA sampling.
Horsman-Hall et al., 2009 [18]	292	Double-swabbing	The Plexor^®^ HY System results proved DNA recovery to be sufficient for STR typing for some samples. When testing a sampling of individuals handling shotshells only as necessary for firing, no significant difference was observed when comparing results obtained from the PowerPlex1 16 BIO and MinifilerTM kits.	Data does not support PCR inhibitors being present in the majority of shotshell case samples, but the results are suggestive that poor STR amplification results in shotshell cases are more likely due to DNA damage, possible degradation, and/or low-level DNA.
Schwender et al., 2021 [60]	168	Single-swabbing	The shedder test results and data ranges were comparable to those of other shedder tests. This study identified moisturisers as a novel factor influencing proposed shedder statuses and corresponding DNA transfer.	To address activity-level hypotheses or questions during legal proceedings, transfer studies with high and low DNA depositors could be executed to encompass a range of possible transfer outcomes.
Jennifer et al., 2009 [101]	252	Double-swabbing	The overall level of DNA recovered from trace samples was quite low.	Considering the large investment in DNA evidence, the relatively simple task may have the potential to greatly increase the resulting number of viable profiles.

## Data Availability

Not applicable.
